# Unveiling the anticancer potentiality of single cell oils produced by marine oleaginous *Paradendryphiella* sp. under optimized economic growth conditions

**DOI:** 10.1038/s41598-023-47656-x

**Published:** 2023-11-26

**Authors:** Hadeel El-Shall, Marwa Abu‑Serie, Gadallah Abu-Elreesh, Marwa Eltarahony

**Affiliations:** 1 Environmental Biotechnology Department, Genetic Engineering and Biotechnology Research Institute (GEBRI), City of Scientific Research and Technological Applications (SRTA-City), New Borg El-Arab City, Alexandria 21934 Egypt; 2 Medical Biotechnology Department, Genetic Engineering and Biotechnology Research Institute, (GEBRI), City of Scientific Research and Technological Applications (SRTA-City), New Borg El-Arab City, Alexandria 21934 Egypt

**Keywords:** Biotechnology, Cancer, Drug discovery, Microbiology, Environmental sciences, Environmental social sciences, Medical research, Oncology

## Abstract

Bioprospecting about new marine oleaginous fungi that produce advantageous bioproducts in a green sustainable process is the key of blue bioeconomy. Herein, the marine *Paradendryphiella *sp. was utilized for single cell oils (SCOs) production economically, via central composite design, the lipid content enhanced 2.2-fold by 5.5 g/L lipid yeild on seawater-based media supplemented with molasses concentration 50 g/L, yeast extract, 2.25 g/L at initial pH value (5.3) and 8 days of static incubation. Subsequently, the fatty acid methyl esters profiles of SCOs produced on optimized media under different abiotic conditions were determined; signifying qualitative and quantitative variations. Interestingly, the psychrophilic-prolonged incubation increased the unsaturation level of fatty acids to 59.34%, while ω-6 and ω-3 contents representing 23.53% and 0.67% respectively. Remarkably, it exhibited the highest EC_100_ dose by 677.03 µg/mL on normal human lung fibroblast Wi-38 cells. Meanwhile, it showed the highest inhibiting proliferation potential on cancer cell lines of A549, MDA-MB 231 and HepG-2 cells by 372.37, 417.48 and 365.00 µg/mL, respectively. Besides, it elevated the oxidative stress, the expression of key apoptotic genes and suppressed the expression of key oncogenes (NF-κB, BCL2 and cyclin D); implying its promising efficacy in cancer treatment as adjuvant drug. This study denoted the lipogenesis capacity of *Paradendryphiella* sp. under acidic/alkaline and psychrophilic/mesophilic conditions. Hereby attaining efficient and economic process under seasonal variation with different Egyptian marine sources to fill the gap of freshwater crisis and simultaneously preserve energy.

## Introduction

Blue biotechnology or marine biotechnology, as most frequently known, endeavors for industrialization and commercialization of marine sources, either marine creatures (crustaceans, sponges, fishes, planktons, microorganisms, etc.) or their bioactive metabolic byproducts. By such way, marine biotechnology contributes substantially in blue bioeconomy^1^. By 2030, blue biotechnology will enhance the entire global economy growth via bioprospecting, which focuses on exploring of new marine biofactories, optimizing the productivity of advantageous metabolites, determining their efficacy, safety testing and ultimately large-scale commercial production of such marine organisms and their metabolic products^2^.

A plethora of marine invertebrates in associations with symbionts microorganisms found their way in recent efficient biotechnological applications, among them fungi^1^. They are accounted for the production of more than 36% of newly discovered natural bioactive molecules, comparing to bacteria which recorded only 14%. Remarkably, oxidative enzymes, hydrolytic enzymes, antibacterial, anti-plasmodial, anticancer, antiviral, anti-inflammatory agents, bio surfactants are categorized among the most widely marine fungal byproducts that find their avenue in multiple applications. In this context^3^, shed the light about the importance of secondary metabolites excreted by marine fungi such as *Penicillium *sp.,* Paradendryphiella *sp.,* Dichotomomyces *sp.,* Neosartorya *sp.,* and Westerdykella *sp. They reported a significant cytotoxicity against different colorectal cancer cell lines. Also, the secondary metabolites of *Aspergillus niger*,* A. oryzae*,* A. fumigatus*,* A. terreus*,* A. flavus* and *A. versicolor* affirmed their effecicncy against cancer cell lines of uterine cervix, colon, ovary and breast^4^. Whereas, the marine ascomycete *Paradendryphiella salina* PC 362H produced secondary metabolite, contained hyalodendrin as the main active ingredient, exhibited antitumor activity in response to MCF7 cell lines and its invasive stem cell-like MCF7-Sh-WISP2 counterpart^5^. Interestingly, other marine fungi (e.g., *Aspergillus *sp.,* Penicillium *sp.,* Cladosporium *sp.,* Lasiodiplodia *sp.,* Rhizopus *sp.* and Mortierella)* isolated from Saudi Arabian mangroves are sources of lipids or single cell oil applied in biofuel production^6^.

Nonetheless, the production of microbial oils or single-cell oils (SCOs) that are abundant with poly unsaturated fatty acids (PUFAs) (e.g., ω-3 and ω-6 classes) from marine fungi is scarcely reported, to the best of our acquaintance, in particular for marine species of *Paradendryphiella*. It is worth mentioning the vital role of PUFAs for human body by the virtue of their anti-thrombotic, anti-irritant and anti-tumor properties^7^. Recently, ω-3 and ω-6 classes of PUFAs gaining a momentum in nutritional and pharmaceutical applications^8^.

Interestingly, the marine fungi exhibit metabolic versatility in their growth conditions and are able to cope high saline conditions of seawater or hypersaline water. Notably, due to water crisis, it becomes indispensable matter to develop competitive alternative non-fresh water processes for reducing the pressure on utilizing freshwater^9^. By employing marine fermentation, seawater will substitute freshwater in media preparation to cultivate marine fungal biomass^10^; besides, avoiding the addition of minerals in the culture media; thereby enhancing the whole economics of the process. Additionally, the overall content of seawater considers being unfavorable for terrestrial and air-born microorganisms. Thus, such content play a selective role against microbial contamination at lab and industrial-scale levels. Furthermore, the employment of non-food or agro-industrial waste as a nutritional alternatives trigger the process more competitive^10^.

Based on the previous backdrop, the main novelty of this work focused on screening and isolation of marine oleaginous fungi with the capability of SCOs production. Besides, economic optimization of SCOs production in an energy preserving process. That was implemented via utilizing agro-industrial waste and non-fresh water under static incubation in statistically design experiments. Further, the fatty acids profile of the extracted SCOs would be determined under different abiotic conditions and their adequacy for employing as antitumor agents would be evaluated as well.

## Results

### Isolation of oleaginous fungi, dry weight determination and quantification of total lipids

**Figure 1:**
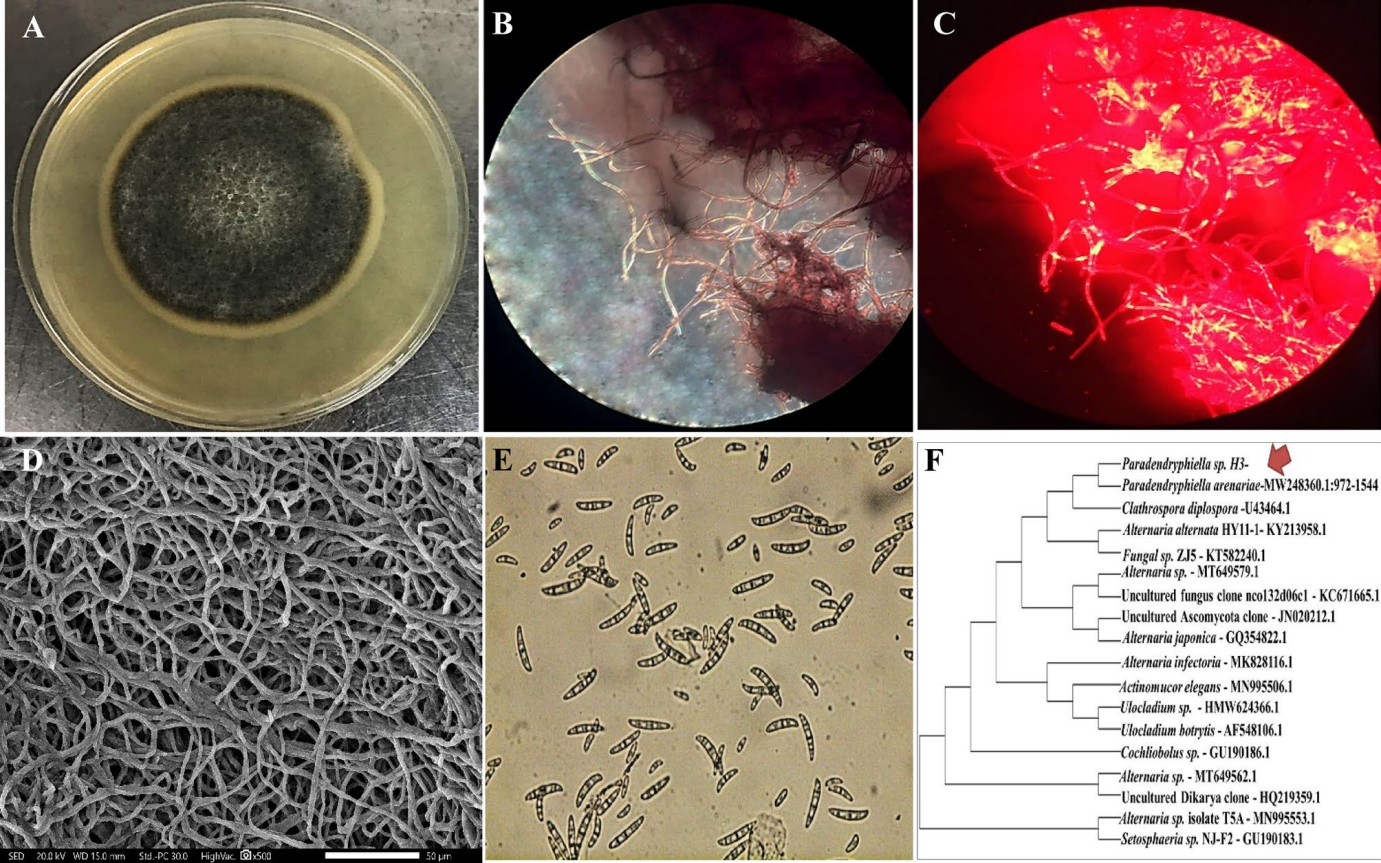
Culture and morphological features of *Paradendryphiella* sp. (**A**) Colony on PDA, (**B**)Fungal hyphae under bright field microscopy (× 100), (**C**) Fungal hyphae contained lipids droplets under fluorescent microscopy of the exact field in (**B**) after Nile-red staining, (**D**) SEM (X500) (**E**) Conidia morphology under light microscope and (**F**) neighbor-joining phylogenetic tree of 18S rRNA gene.

A total of 23 marine fungal isolates were screened from Mediterranean Sea and examined for their lipid accumulation ability qualitatively by Nile-red staining technique (NR). Based on fluorescence microscope inspection, five fungal isolates showed many oval and ellipsoidal fat globules as red oil droplets and signified as potential lipid producers (Fig. 1). Remarkably, NR assay and fluorescence microscopy deemed as preliminary, fast and effective tool to distinguish oleaginous fungi from non-oleaginous^11^. Thereafter, the isolates with strong red fluorescence signals were selected for lipid extraction in lipid production broth. After 7 days of incubations the dry cell weight, lipid yield and lipid content were quantified as shown in Supplementary Table S1. The results obviously indicated that the tested isolates had the ability to accumulate lipids in different amounts ranging from 0.85 to 2.61 g/L of their dry weight. Thereby, the isolate symbolized as H3 was picked up for further study stages. As it recorded the strongest lipid producer, among the screened marine oleaginous fungi, by its highest lipid yield (2.61 g/L) and lipid content (22%).

### Morphological characterization and molecular identification of the selected fungal isolate

The selected isolate exhibited good growth on peptone dextrose agar (PDA) plates and its colonies showed blackish to olivaceous-black color with floccose like texture and surrounding with white edges (Fig. 1A). The morphological features, which were visualized under light microscope (Fig. 1B,C) and scanning electron microscope (SEM) (Fig. 1D), displayed hyaline, smooth, simple, and branched hyphae with diameter ranged from 2 to 7 μm either solitarily or in groups. The conidia appeared cylindrical or sub-ellipsoidal with 3 to 6 septa (Fig. 1E). However, the taxonomic identification inquiry through 18S rRNA gene sequencing indicated 99% similarity with *Paradendryphiella *sp. as revealed by Blastn analysis; submitted in NCBI GenBank under accession number of OQ134928. The Neighbour-joining (NJ) approach was applied to construct the phylogenetic tree of the strain under study as illustrated in (Fig. 1F).

### Optimization of nutritional and incubation parameters

#### Central composite design (CCD)

The present study demonstrates the merits of employing the recent statistical optimization approaches in terms of energy saving (static conditions), seawater-based aqueous medium (instead of fresh water) and low-cost nutritive substrate (agro-industrial waste or molasses) for economic feasibility of SCOs production. Initially, the effect of static and shaking (150 rpm) incubation in lipid accumulation was screened (data not shown). The results revealed insignificant difference between both conditions, despite superior productivity under shaking. The lipid content recorded 22.08% under shaking; however, it reached 21.9% under static. Subsequently, the all trials of CCD were incubated statically. Herein, thirty-one experimental trials with different combinations of initial pH level, incubation time (days), molasses concentration and yeast extract concentration were investigated. As observed in Table 1, the different coded and actual levels of the four independent parameters and the lipid yield (g/L) as a response in each run were illustrated. The results demonstrated a considerable variation in the lipid yield, which recorded the maximum value with 5.899 g/L in trial 15, at pH level (5), incubation time (7 days), molasses concentration (50 g/L) and 2 g/L of yeast extract concentration. Whereas, the minimum yield was obtained by 0.079 g/L at 4th run with 30 g/L of molasses, 2 g/L of yeast extract, incubation time (7 days) and pH level (3).

**Table 1:** Central composite design matrix of actual lipid yield generated from marine oleaginous fungi *Paradendryphiella *sp. influenced by yeast extract concentration, molasses concentration pH and incubation time along with the predicted responses and standardized residuals.

Run order	Molasses (g/L)	Yeast extract (g/L)	Incubation time (day)	pH	Experimental lipid weight (g/l)	Predicted lipid weight (g/l)	St. residual
1	1	1	1	− 1	2.451	2.571	− 0.65
2	1	1	− 1	1	2.377	2.606	− 1.24
3	1	− 1	− 1	1	2.611	2.485	0.68
4	0	0	0	− 2	0.079	− 0.102	0.98
5	1	− 1	1	1	2.305	2.609	− 1.65
6	0	0	0	0	2.862	2.462	1.51
7	0	0	2	0	0.736	0.819	− 0.45
8	0	0	0	0	2.119	2.462	− 1.3
9	0	0	0	0	2.464	2.462	0.01
10	− 1	1	1	− 1	1.095	1.288	− 1.05
11	0	− 2	0	0	1.624	1.62	0.02
12	0	0	0	2	0.946	0.902	0.24
13	0	0	0	0	2.038	2.462	− 1.6
14	− 1	1	− 1	− 1	0.753	0.607	0.79
15	2	0	0	0	5.899	5.599	1.63
16	− 1	− 1	− 1	− 1	0.978	1.107	− 0.7
17	− 1	− 1	1	− 1	0.951	0.88	0.38
18	1	1	− 1	− 1	0.648	0.883	− 1.28
19	0	0	− 2	0	0.322	0.014	1.67
20	− 1	1	1	1	1.794	1.682	0.61
21	− 1	− 1	− 1	1	1.006	1.044	− 0.21
22	− 1	1	− 1	1	1.447	1.658	− 1.14
23	0	0	0	0	2.46	2.462	− 0.01
24	− 1	− 1	1	1	0.33	0.161	0.91
25	0	2	0	0	2.37	2.149	1.2
26	− 2	0	0	0	2.8	2.875	− 0.41
27	1	− 1	− 1	− 1	1.606	1.876	− 1.46
28	1	1	1	1	3.7	3.637	0.34
29	1	− 1	1	− 1	2.8	2.656	0.78
30	0	0	0	0	2.701	2.462	0.9
31	0	0	0	0	2.589	2.462	0.48

Variable	Coded levels/experimental values						
	− 2	− 1	0	1	2		
Molasses (g/L)	10	20	30	40	50		
Yeast Extract (g/L)	1	1.5	2	2.5	3		
Incubation time (day)	4	6	7	8	10		
pH	3	4	5	6	7		

### Regression and analysis of variance (ANOVA)

The multiple regression analysis was employed to statistically analyze the data of lipid yield by *Paradendryphiella *sp. as tabulated in Table 2, which also included the values of coefficient of determination (R^2^), the adjusted-R^2^ (Adj-R^2^), the coefficient estimates as well as probability *P*-value, lack-off fit, linear, quadratic and interactions impacts. As noticed, the value of R^2^, which determines the effectiveness of the polynomial regression model, assessed by 0.961 which proves 96.1% of variation in lipid yield was impacted by the independent variables and only 3.9 could not be explained in the view of model. Generally, the model was considered being strongly correlated at R^2^ value more than 0.9^12^. Besides, the Adj-R^2^ value was quantified as 0.928, which emphasized the model significance. Notably, the small difference between R^2^ and Adj-R^2^ reflects the good coordination between the actual experimental values and the predicted values of lipid yield; thus, the model of the current study is optimal within the range of experimental factors to predict an efficient lipid yield. In addition, the positive coefficient values pointed out that the linear effect of all variables, quadratic effect of molasses and mutual interactions effect of some factors exhibited synergistic effect in lipid yield (i.e., their higher values enhance lipid yield). While the other factors, which displayed negative coefficient values signifies their higher impact on lipid yield at their negative values.

**Table 2:** Estimated effects, regression coefficients and corresponding *P-*values for second order polynomial model of lipid yield extracted from *Paradendryphiella* sp. and optimized by CCD.

Term	Coef	SE Coef	T	*P*
Constant	2.4619	0.10801	22.793	0
Linear effects				
Molasses	0.6809	0.05833	11.673	0
Yeast extract	0.1321	0.05833	2.264	0.038
Incubation time	0.2012	0.05833	3.449	0.003
pH	0.2509	0.05833	4.302	0.001
Quadratic effects				
Molasse*Molasse	0.4438	0.05344	8.305	0
Yeast extract*yeast extract	− 0.1443	0.05344	− 2.701	0.016
Incubation time				
*Incubation time	− 0.5113	0.05344	− 9.569	0
pH*pH	− 0.5155	0.05344	− 9.646	0
Interaction effects				
Molasse*yeast extract	− 0.1231	0.07144	− 1.723	0.104
Molasse*Incubation time	0.2517	0.07144	3.524	0.003
Molasse*pH	0.168	0.07144	2.352	0.032
Yeast extract * Incubation time	0.2269	0.07144	3.176	0.006
Yeast extract*pH	0.2784	0.07144	3.897	0.001
Incubation time *pH	− 0.164	0.07144	− 2.296	0.036
R-Sq = 96.9%	R-Sq(adj) = 94.02%			

The ANOVA data of the model was calculated for lipid yield and unveiled the highly significance of the model; assuring by a very low *P*-value, which quantified as 0.000 (Supplementary Table S2). It is noteworthy mentioning that the *p*-value evaluated the significance of each variable and simultaneously identified the effect of each factor on the response. Namely, the parameters with *P*-values ≤ 0.05 are deemed having statistically significant impacts and the factors with *P*-values exceed 0.05 are statistically nonsignificant^13^. Hence, the interaction effect of molasses and yeast extract is nonsignificant as its corresponding *P*-value recorded 0.104 (Table 2). Seemingly, as deduced from Fisher’s F test (ANOVA, Supplementary Table S2), the quadratic influence of the examined factors showed more predominance in improving lipid yield followed by linear and interaction impacts. Eventually, the coefficients were fitted to the second-order polynomial equation for expressing the correlation between dependent response (i.e., lipid yield) and independent examined factors (Eq. 1).

1 $$ \begin{aligned} & {\text{Lipid}}\;{\text{yield}} = {2}.{46} + 0.{68}\;{\text{Molasses}} + 0.{132}\;{\text{Yeast}}\;{\text{extract}} \\ & \quad + 0.{2}0{1}\;{\text{Incubation}}\;{\text{time}} + 0.{25}\;{\text{pH}} + 0.{44}\;\left( {{\text{Molasses}}} \right)^{{2}} \\ & \quad {-}0.{144}\;\left( {{\text{Yeast}}\;{\text{extract}}} \right)^{{2}} {-}0.{511}\;\left( {{\text{incubation}}\;{\text{time}}} \right)^{{2}} \\ & \quad {-}0.{515}\;\left( {{\text{pH}}} \right)^{{2}} {-}0.{123}\;{\text{Molasses}}*{\text{yeast}}\;{\text{extract}} \\ & \quad + {\text{Molasses}}*{\text{Incubation}}\;{\text{time}} + 0.{168}\;{\text{Molasses}}*{\text{pH}} \\ & \quad + 0.{226}\;{\text{Yeast}}\;{\text{extract}}*{\text{Incubation}}\;{\text{time}} \\ & \quad + {\text{Yeast}}\;{\text{extract}}*{\text{pH}}{-}0.{164}\;{\text{Incubation}}\;{\text{time}}*{\text{pH}} \\ \end{aligned} $$

### Graphical demonstrations of the response surface model

**Figure 2:**
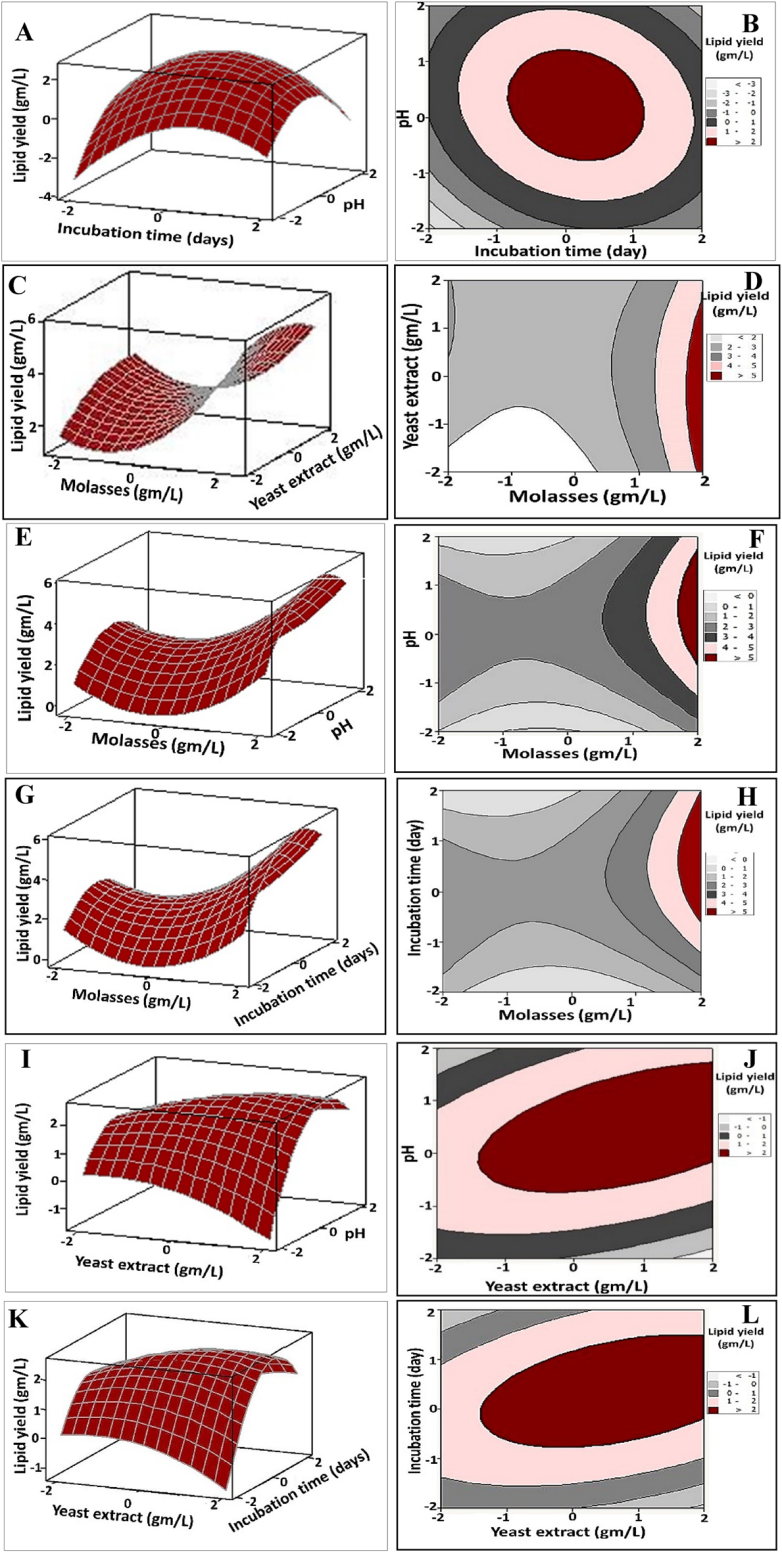
Three-dimensional surface and two-dimensional contour plots for lipid yield (g/L) produced by *Paradendryphiella* sp., displaying the interactive effects of the molasses concentration, yeast extract concentration, pH and incubation time. The plots were displayed by Minitab 15.0.

The three-dimensional (3D) surface and two-dimensional (2D) plots were generated to demonstrate the interaction effect of pairwise combination of the selected independent parameters on lipid yield that was represented on the z-axis, while the other examined parameters were kept at their central level (Fig. 2). Besides, such plots give an insight about the optimal conditions for the maximum productivity of lipids by *Paradendryphiella *sp. Figure 2A,B depicted the lipid yield as a function of incubation time and pH; showing that the maximum lipid yield was obtained at the middle levels of both variables (i.e., pH (4–6) and incubation time (6–8 days). While, Fig. 2C,D reflected the antagonistic effect between yeast extract and molasses concentrations on lipid yield. Wherein, the maximum lipid yield was achieved at the highest molasses concentration (i.e., 50 g/L) with lower concentrations of yeast extract. On the other hand, the initial concentration of molasses up to 50 g/L exhibited a steady positive effect on the lipid yield at neutral values of pH (Fig. 2E,F). In the same vein, by analysis of Fig. 2G,H and solving the Eq. (1), the maximum predicted lipid yield (more than 5 g/L) could be reached by utilizing 50 g/L of molasses and prolong the incubation time to 10 days. Apparently, a significant synergistic effect could describe the interaction effect between yeast extract concentration & pH (Fig. 2I,J) and yeast extract concentration & incubation time (Fig. 2K,L) on lipid yield. Wherein, more than 2 g/L of lipid could be obtained by using yeast extract concentration in the range of 1.5 to 3 g/L within 6–8 days at neutral pH ranges.

### The desirability function for prediction of the optimum conditions and model validation

The substantial aim of the statistical design of experiment focuses on attaining the maximum lipid yield by marine oleaginous *Paradendryphiella *sp. in the terms of optimum levels of examined variables. The optimum predicted values and the desirability function for the maximum productivity of lipid yield depicted in Supplementary Fig. S3. The optimum working conditions were initial pH value (5.3), molasses concentration 50 g/L, yeast extract, 2.25 g/L under 8 days incubation time, which resulted in 5.8746 g/L of lipid, with desirability value recorded 0.99580. In order to verify the lipid yield under the former predicted conditions and also to evaluate the optimized conditions versus the original medium before optimization, the experiments were carried out in triplicates for each trial. The results highlighted that lipid yield obtained from optimized conditions were 5.5 g/L with 40.7% lipid content; reflecting a perfect correlation between the actual values and predicted ones. Intriguingly, there is about 2.2-fold increases in lipid yield and lipid content comparing to the basal medium which recorded 2.5 g/L and 20.16%, respectively.

### Fatty acid composition profile under optimized conditions and different abiotic factors

This stage was implemented to determine the influence of different abiotic factors (i.e., temperature, pH and incubation time) on the profile composition and their adequacy for subsequent antitumor application. On optimized media composition and under different abiotic conditions, SCOs produced by oleaginous marine *Paradendryphiella *sp. was extracted. The fatty acid methyl esters profiles that were generated after acidic catalyzed transesterification of lipids revealed both qualitative and quantitative discrepancies among all examined profiles (Table 3). The profile of lipids produced after one month incubation at 10 °C exhibited the prevalence of unsaturated fatty acids (USFAs) by 59.34%. Otherwise, saturated fatty acids (SFAs), mainly C18 and C16 FAs, were the dominate constituent in the profiles of all other conditions. The gas chromatography (GC) analysis results of all profiles showed a common feature among lipid profiles of all abiotic conditions. As Palmitic acid-C16, which is among SFAs, was found being present in the highest amount ranged from 21.63 to 58.18%, followed by Oleic acid -C18 monounsaturated fatty acids (MUSFAs) which oscillated in its value from 1.62 to 37.96%; however, the third major constituent was Stearic acid-C18 (SFAs) with values recording 5.25% to 20.29%. On the other hand, an obvious enhancement in the quantity of some FAs was observed upon prolonged incubation. As noticed the content of ω-6 increased from 6.97 to 14.09 and 23.53% upon extending incubation time from 8 days to one month either at 28 °C or 10 °C, respectively. In the same sense, a notoriously elevation in the content of ω-3 from 0.18 to 1.94% and 0.67% at the exact conditions. Interestingly, the appearance of new components of FAs was also shown at incubation under low temperature, particularly in long chain FAs, including behenic acid (C22), tricosanoic acid (C23), lignoceric acid (C24). Besides, considerable percentages of pentadecanoic acid (C15), margaric acid (C17), arachidic acid (C20) were also detected, which all belongs to SFAs. Additionally, ω-9 (Erucic acid and Cis-11- Eicosanoic acid; C22), which categorizes to MUSFAs was noticed in the profile of lipids extracted from biomass incubated mesophilically at one month with 2.32%; however, it was undetected in psychrophilic incubation. Notably, the lipid profiles under acidic and alkaline conditions exhibited the disappearance of some components and the prevalence of only Palmetic acid-C16 (SFAs), Oleic acid -C18 (MUSFAs) and Stearic acid-C18 (SFAs), which could be invested posteriorly as ω-7 supplement.

**Table 3:** Fatty acids composition of lipids produced by *Paradendryphiella *sp. under optimized conditions and other abiotic factors.

Fatty acid (FA) name	FA type	FAs %-optimized	FAs %-optimized-10 °C	FAs %-28 °C -1 months	FAs %-10 °C -1 months	FAs% pH 3	FAs% pH 9
Tetradecanoic acid	SFA	0.11	0.3	2.84	3.50	1.73	–
Pentadecanoic acid	SFA	–		2.15	1.88	0.35	–
Hexadecanoic acid	SFA	39.00	40.05	21.36	22.89	50	58.18
Heptadecanoic acid	SFA	–		1.41	–	–	–
Octadecanoic acid	SFA	12.16	11.16	5.47	5.25	9.45	20.29
14-Hydroxy-14 methyl hexadec-15-enoic acid	SFA	–		4.33	–	–	–
Octadecanoic acid 9,10-Dihydroxy	SFA	3.91	4.01	10.30	–	14.30	18.70
Eicosanoic acid	SFA	–	–	2.44	3.00	–	–
Docosanoic acid	SFA	–	–	1.53	1.42	–	–
Tricosanoic acid	SFA	–	–	0.91	0.82	–	–
Tetracosanoic acid	SFA	–	–	1.64	1.90	0.14	–
6,9,12,15- Docosatetraenoic acid	SFA	0.25	0.19	–	–	–	–
Docosanoic acid	SFA	0.05	0.08	–	–	–	–
Tetracosanoic acid	SFA	0.04	0.07	–	–	–	–
9-Hexadecenoic acid	MUFA	0.53	0.71	9.35	8.53	3.40	–
Erucic acid	MUFA	–	–	–	1.35	–	–
Cis-10-Heptadecenoic acid	MUFA	–	–	1.43	1.33	–	–
9-octadecenoic acid	MUFA	36.80	37.96	18.81	22.05	17.00	1.62
Cis-13-Eicosenoic acid	MUFA	–	–	–	0.91	–	0.77
Cis-11- Eicosanoic acid	MUFA	–	–	–	0.97	–	–
9,12-octadecadienoic acid	PUFA (omega6)	6.70	5.37	10.56	17.16	3.34	–
11,14-Eicosadienoic acid	PUFA (Omega6)	0.02	–	2.44	0.91	0.31	0.44
6,9,12- Octadecatrienoic acid	PUFA (Omega6)	0.25	–	1.09	5.46	–	–
9,12,15-octadecatrienoic acid	PUFA (Omega3)	0.18	0.10	1.94	0.67	–	–
SFAs%		55.52	55.86	54.38	40.75	75.97	97.17
USFAs%		44.48	44.14	45.62	59.34	24.05	2.83
MUFAs%		37.33	38.67	29.59	35.14	20.4	2.39
PUFAs%		7.15	5.47	16.03	24.20	3.65	0.44
Omega 3%		0.18	0.10	1.94	0.67	–	–
Omega 6%		6.97	5.37	14.09	23.53	3.65	0.44

### Determination of cytotoxicity and anticancer activity of SCOs on normal human and cancer cell lines

**Figure 3:**
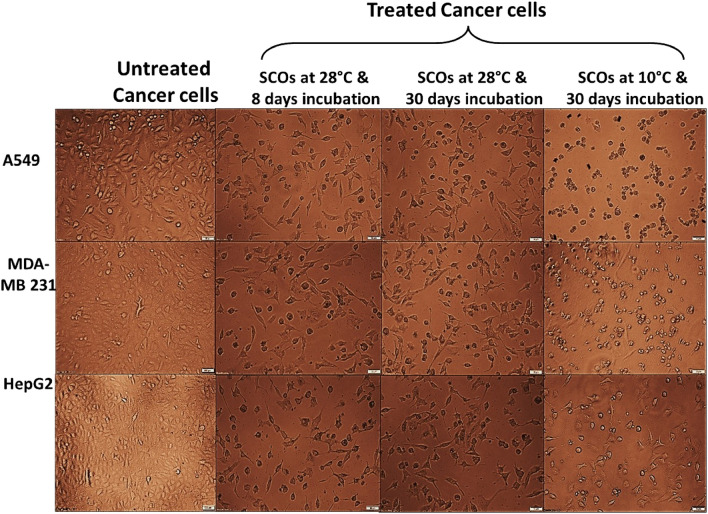
Morphological alteration of the SCOs-treated human cancer cells in comparison with the untreated control cancer cells.

Based on the FAs composition of SCOs produced by *Paradendryphiella *sp., the SCOs produced under optimized and prolonged incubation at mesophilic and psychrophilic conditions were selected for evaluating their anticancer activity. Firstly, the influence of SCOs on the viability of normal human cells was assessed via determining the safe doses (EC_100_) of SCOs that attained 100% viability of normal human lung fibroblast Wi-38 cells. As shown in Table 4 the highest EC_100_ value of SCOs produced under optimized and cold prolonged conditions; referring to their highest safety on the proliferation of normal human cells up to 602.02 and 677.03 µg/mL, respectively. Besides, SCOs produced under prolonged psychrophilic conditions exhibited the highest inhibiting proliferation potential on A549, MDA-MB 231 and HepG-2 cells by 372.37, 417.48 and 365.00 µg/mL, respectively, in dose-dependent manner (Table 4, Supplementary Fig. S4). Noteworthy mentioning that the anticancer activity was determined through measuring half maximal inhibitory concentration (IC_50_), which evaluates the strength of SCOs in inhibiting 50% of cancer cells growth and their biochemical function. Where the lowest IC_50_ value is an indicator of the strongest anticancer activity. Furthermore, to visualize the cellular morphological alterations induced by the treatment of SCOs, phase contrast inverted microscope was used. As observed in Fig. 3, the untreated cells appeared healthy maintaining their normal spindle shape; however, upon treatment, a whole collapse was observed indicated by cell shrinkage.

**Table 4:** The half-maximal inhibitory values (IC_50_) of the studied SCOs on human cancer cells and their safe doses (EC_100_) on normal cells.

Sample	IC_50_ (µg/mL)			EC_100_ (µg/mL)
	A549	MDA-MB 231	HepG2	
SCOs at 28 °C and 8 days incubation	660.52 ± 21.79^c^	841.29 ± 5.053^c^	696.15 ± 1.31^c^	602.05 ± 7.98^a^
SCOs at 28 °C and 30 days incubation	472.49 ± 21.58^b^	538.63 ± 8.26^b^	516.74 ± 9.12^b^	356.89 ± 28.74^b^
SCOs at 10 °C and 30 days incubation	372.37 ± 22.85^a^	417.48 ± 10.54^a^	365.00 ± 25.07^a^	677.03 ± 13.31^a^

All values are expressed as mean ± SEM. Different letters (a, b, and c), for the same parameter (IC_50_ of each cancer cell line and EC_100_), are statistically significant between extracts at *p* < 0.05 using the one-way ANOVA test with Tukey-Post Hoc’s multiple comparison. These letters refer sequentially to extract values from the lowest IC_50_ or the highest IC_100_ to the highest IC_50_ or the lowest IC_100_, respectively.

### Effect of SCOs on cellular lipid peroxidation

The oxidative stress exerted by all examined SCOs was determined through estimating the lipid peroxidation. Notably, the SCOs produced under psychrophilic/ prolonged incubation showed the highest potential in elevating cellular lipid peroxidation in all treated cancer cell lines by ≥ 4 folds, compared to less than 3 folds and 5 folds for SCOs produced at 28 °C either after 8 or 30 days, respectively (Supplementary Table S5).

### Impact of SCOs on the expression of key oncogenes and proapoptotic genes

Accordingly, the most potent anticancer activity was clearly evident against all cancer cell lines treated with SCOs produced under psychrophilic/ prolonged incubation. Therefore, it was subjected to additional mechanistic research through examining the expression of key oncogenes (NF-κB, BCL2 and cyclin D). Supplementary Table S6 declared that SCOs produced under psychrophilic/ prolonged incubation suppressed the expression of all examined key oncogenes by more than 4 folds relative to the untreated A549 cancer cells. Moreover, it can upregulate the expression of proapoptotic genes (p53 downstream genes, including Bax and p21) by 3.72–5.35 folds compared to the untreated A549 cells.

## Discussion

In the recent decades, microbial oils or SCOs, are deemed as the most promising feedstock for biofuel and nutraceuticals production for their structure vicinity with fish oil or even vegetable oils^14^. Remarkably, oleaginous fungi had gained a momentum in lipid production by the virtue of their short growth cycle, large quantity of biomass, high lipids yield and facile collection under fermentation conditions^15^, which all participate in enhancing techno-economic productivity. Notably, the majority of studies targeting mycological production of SCOs are terrestrial, wastewater and fresh water origin^16^. However, the marine ecosystems characterize by their wide diversity with broad environmental gradients in chemical, physical, and hydrological parameters, like pH, high salinity, high osmosis, low temperature, light intensity and high pressure. In a consequence, their dwellers of marine microorganisms exhibit different adaptability scenarios, to cope with such diverse stressors they exposed, comparing to their terrestrial counterparts^17^. Therefore, the current research focused on mycological production and optimization of lipids from marine sources in an economic process without consuming freshwater and other expensive nutritional source and in an adequate frame of time. Besides, determining its profile under different conditions and evaluating their anticancer potentiality.

Initially, five fungal isolates showed intracellular ellipsoidal fat globules as visualized by NR staining under fluorescence microscope. As NR dye is a fluorescent lipophilic probe with great fluorescence emission spectrum, so, it was used to detect these intracellular lipid bodies or SCOs in intact cells^18^. While the number, size and shape of lipid bodies could give quick insight about the potency of microorganism in lipid production^19^. Only, the isolate that displayed the highest quantity of lipid was identified molecularly as *Paradendryphiella *sp. It belongs to Ascomycota, which were reported as potent lipid producers^20^. Productions of SCOs from fungal sources were obviously targeted to Ascomycota especially of the genera *Aspergillus*^16^, *Lasiodiplodia*,* Phomopsis*, *Pestalotiopsis* and *Phomopsis*^20^.

Since the nutrients are consider being the most fundamental parameter that governing the biochemical performance of microbes and improving or lowering their metabolites productivity, the subsequent stage aimed to maximize lipid content by *Paradendryphiella *sp. through adjustment of process inputs, which including nutrients and incubation conditions without elevating the process cost. It was achieved by employing different intended aspects. It commenced firstly by determining the influence of static/shaking incubation on lipid content, which indicated a nonsignificant difference. Therefore, the static incubation was fixed during all study trials; despite shaking incubation is crucial factor in microbial growth and metabolites production^21^. Notably^22^, found that lipid accumulation by *Aspergillus *sp. strain EM2018 reached 32.7 and 27.6% lipid/dry biomass in static and shaking conditions, respectively after 7 days of incubation, attributing this result to the uplifting in saturated fatty acids quantity (the basal ingredient of lipids) due to the diminishing in the amounts of phospholipids and sterols and the alterations in the glyceride fraction. Conversely^23^, reported that *Yarrowia lipolytica* yielded higher lipid content when incubated at 150 rpm by 23.6% rather than static incubation by 17.5%; assigning that to the role of aeration degree in enhancing fungal biomass and consequently lipid content. Secondly, utilizing seawater instead of freshwater as an aqueous medium; therefore, averting the supplementation with minerals and trace elements in the culture broth. The importance of addition inorganic salts supplementation of K, Na, Mg, Cl, S, P and Zn in elevating fungal growth and lipids productivity by *Cryptococcus laurentii* was stated by^24^.

Thirdly, as commonly known, the production cost of any fermentation process and subsequent SCOs cost were calculated from the carbon source or nutritional substrates^25^. Hence, utilizing agro-industrial wastes such as molasses, as a carbon source, seems to be more cost-effective process. Molasses is dark brown viscous liquid generated from cane or beet sugar processing industries as a by-product. It was reported extensively as one of the remarkable nutritive feedstock for the production of SCOs^26^, lactic acid^27^, bio-surfactant, bio-polymers^28^ and biodiesel^29^. Thus, the implementation of our study under such design was considered being economically competitive relative to the majority of microbial-based marine biotechnology processes^9^. It avoided using of high energy- requirement in energy-intensive cultivation processes (i.e., agitation), excessive consumption of fresh water especially at industrial scale and condoned the consumption of expensive nutritive resources.

In this context, the finding out of exact amounts of nutrients and accurate environmental condition was undertaken and performed through statistical design of experiment. This strategy recognized by saving time, efforts, and tools; perceive considerably the interaction effects among examined parameters, which was neglected by single dimensional approach or one variable at time (OVAT). Via CCD method, lipid content of *Paradendryphiella *sp. were assessed in 31 trials with gradual levels of pH, molasses concentration, yeast extract concentrations during 4–10 days as incubation time. The results revealed that *Paradendryphiella *sp. required 50 g/L of molasses, 2.25 g/L of yeast extract at 5.3 as pH value and 8 days incubation time to ameliorate lipid content 2.2-fold by 40.7%, relative to basal medium (20.16%). It is worth mentioning the nutritive value of molasses, as it comprises uncrystallized sugar, glucose, fructose and sucrose that exceeded 60% of its sugar content^26^. Let alone other ingredients such as organic acids (≈6%), vitamins, proteins (≈13%), minerals (11.7%) and other phenolic compounds as revealed by ^26^. However, the appropriate molasses concentration favors lipogenesis process, otherwise, inadequate dose is channeled toward fungal biomass formation and polysaccharides storage^30^. Nonetheless, the toxic effect of molasses on microbial growth and lipid synthesis upon elevating molasses concentration to 15 g/L was reported by^26^. He and coauthors attributed this result to the osmotic pressure increment at higher molasses concentration, which contradict our finding. Also, the oversupply of carbon source (60 g/L) lessened lipid content of *R*,* mucilaginosa* as found by^31^. Intriguingly, marine origin of oleaginous *Paradendryphiella *sp., under study, assisted not only in the tolerance of osmotic pressure generated from molasses concentration but also in converting sugar content into lipids. In the same extend^25,30^, stated that the higher synthesis of lipids by *Rhodotorula glutinis TR29* and *Zygosaccharomyces siamensis AP1*was obtained upon incorporating 20% molasses in fermentation media, which harmonized with our results. Meanwhile^26,32^, combined other supplements to molasses such as glycerol (30 g/L) to boost lipid content produced by *Yarrowia lipolytica* and *Rhodotorula glutinis* R4, which seemed to be additional cost.

Regarding the nitrogen source, it is another limiting parameter that manages cellular growth and subsequent lipid accumulation. As referred by^22^, the metabolic byproduct of nitrogen source is the responsible for altering the flux of carbon to the production of lipid precursors. Particularly, the concentration, the source of nitrogen, organic, inorganic or complex and even its ratio to carbon source contribute to great extent in lipogenesis process^33^. Where ammonium sulfate improved lipid yield of *Mucor plumbeus* from 2.44 ± 0.01 to 3.62 ± 0.03 g/L, comparing to organic sources (yeast extract, peptone, casein and malt extracts), which ranged from 1.48 ± 0.01 to 2.83 ± 0.01 g/L^34^. Likewise, the highest lipid production (3.03 g/L) was obtained by *Fusarium oxysporum* after the addition of ammonium phosphate^33^. Generally, the ammonia salts are the most frequently utilized nitrogen source in lipid production^25^. On the other hand^22,35^, unveiled that terrestrial fungi *Mucor *sp. and *Aspergillus *sp.* strain EM2018* preferred complex nitrogen source (e.g., peptone, meat extract, yeast extract and malt extract) more than inorganic source in increasing the productivity of lipid (≥ 50%); referring to the superiority of yeast extract due to its easy solubility and readily availability. Nevertheless, other investigations reported the reinforcement role of merging between organic and inorganic nitrogen sources or even more one than organic sources of nitrogen in improving lipid accumulation^35^, which deemed as uneconomic. Noteworthy giving an insight about the importance of nitrogen starvation relative to the carbon (i.e., higher C/N ratio), that causes inactivation of key-enzymes contribute in acetyl-CoA production, which is further assimilated to fatty acids and finally triglycerides^36,37^. Broadly, the most convenient C/N ratio is ˃ 20 as denoted by^25,33,38^. Herein, the C/N ratio assessed by 22.2, which imitated with that reported by^30^.

Remarkably, the hydrogen ion concentration or pH of the medium is accountable for the regulation of plasma membrane function in maintaining membrane osmosis to absorb or transport of certain ions in and out the cells^21^. Let alone its influential role in the overall metabolic pathway with catalyzing enzymes and the entire biochemical performance of the cells. Under a slightly acidic to neutral pH, the optimal growth of fungal cells is detected. However, the most prober pH for biomass formation and lipid bodies synthesis was reported at 5 as revealed by^22^. In this extent^39^, manifested that *Penicillium brevicompactum* NRC accumulated maximum lipid content at pH 5, which was consistent with the data of the current study. Whereas^40^, pointed out that the lipid content (62.4%) produced by *Trichosporon fermentans* at pH 6.5 after 7 days of incubation. Otherwise^41^, reported the capability of *Rhodosporidium* TJUWZ4 and *Cryptococcus* TJUWZA11 to exhibit the maximum lipid yield (more than 44%) at pH 4. Broadly, the improper pHs cause insufficient growth followed by low lipid content due to their drastic effect on the metabolic functionality of the enzymes involved in lipid production pathway^37^. On the other hand, enough incubation time allows lipid accumulation sufficiently with good exhaustion of nutrients and before consuming the stored lipids as an energy source, after the depletion of available nutrients^37^. Herein, 8 days as incubation period seemed to be characteristic comparing to investigation conducted by^24^, who declared that extending incubation time from 240 to 360 h was essential to increase lipids production by *Cryptococcus laurentii*. The results of the present study approximated the finding of other studies who documented that 4 and 5 days of incubation were enough to maximize lipid productivity by *Aspergillus *sp., and *Trichoderma viride* NRC314, respectively; implying strain specificity^22,37^.

Accordingly, it is plausible stating that lipogenesis, as a dynamic process, and subsequent fatty acid profile mainly rely on physiological behavior of the oleaginous microbe and abiotic factors, which including nutritional substrates, their quantities, initial pH, incubation time, cultivation temperature, dissolved oxygen and agitation state^34^. In the same scope and in the avenue of economic productivity, the influence of different temperatures (i.e., 10 °C and 28 °C), pH and prolonged incubation time (i.e., 30 days) on lipid accumulation and fatty acid profile of lipid extracted from oleaginous *Paradendryphiella *sp. was determined. Broadly, various fatty acids with carbon backbone of C14 to C24 were produced. This result came in a harmony with^42^ who reported that fatty acid profile of fungal biomass was mainly palmitic followed by oleic and linoleic fatty acids. The fatty acids composition of the present study is quite similar to SCOs extracted from *Aspergillus terreus* isolated from mangrove wetland with dominance of palmitic acid in the range of 20–36% followed by Stearic acid in the range of 20–23%^19^. Obviously, the alteration in pH values from acidic to alkaline, led to chief modifications in their FAs compositions both qualitatively and quantitatively. The predominance of SFAs (e.g., Palmetic acid and Stearic acid) with range of 91–99% directed us to future investment of lipids generated from extreme pH conditions in insulin sensitivity, cholesterol metabolism, anti-thrombosis, β-cell apoptosis preventing and wound healing as well^43,44^.

Intriguingly, our study unveiled the capability of marine oleaginous *Paradendryphiella *sp. to accumulate lipids under psychrophilic and mesophilic conditions with marginal difference in lipid yield and obvious variation in their composition. That could be attributed to the adaptive response adopted by *Paradendryphiella *sp. by the virtue of its marine origin. Such inherit property enabled the oleaginous filamentous fungus to increase the degree of FAs unsaturation to maintain and regulate the fluidity of the membrane under cold stress conditions as recorded by^45^. Similarly^46^, recorded the emergence of stearidonic acid (C18:4) in *Mortierella *sp. under low temperature. Additionally^30^, found the exact observation for *R. glutinis* TR29 when incubated under 10 °C and 15 °C. Conversely^47^, recorded higher similarity in FAs profiles of lipids extracted from *Scenedesmus* sp. Z-4 that was incubated at 10, 15, and 25 °C. He and his coworkers found a gradual decrease in biomass, lipid content and lipid productivity at lower temperatures; assigning this result to the inhibition of enzymatic system responsible for cell growth and lipid accumulation and also inefficient substrate consumption at cold conditions.

Notably, the elevation of ω-6 and ω-3 contents of SCOs produced by *Paradendryphiella *sp. was noticed upon prolonged incubation up to one month, which seemed to be advantageous. Other studies^48,49^ recorded the absence or even limited amount of PUSFAs in the lipid profile of *Rhodococcus opacus* and *Aspergillus caespitosus*, respectively. Generally, our finding is consistent with the results obtained by^23^, who recorded the presence of ω-6 PUSFAs, ω-7 PUSFAs and ω-9 MUSFs by 2.66, 2.74 and 5.68%, respectively after incubating the *Yarrowia lipolytica*Y4 for one month, while, the profile after 7 days incubation generated ω-3 PUSFAs and ω-9 MUFA by 2.82 and 10.88%, correspondingly. Remarkably^50^, reported that the dominance of Oleic acid (C18:1n9) content with lower SFAs content always determine the quality of SCOs which directed toward fuel applications as biodiesel.

However, the FAs fractions of SCO synthesized by marine *Paradendryphiella *sp. of the present study make it more adequate for human use either nutritionally, medically or pharmaceutically. Several biological activities were documented for heptadecanoic, pentadecanoic acid, arachidic acid, behenic acid, tricosanoic acid and lignoceric acid in enhancing insulin sensitivity, reducing risk of diabetes / cardiometabolic diseases, slowing the progress of alzheimer disease, enhancing cellular membranes flexibility in the nervous system/ skeletal muscle/immune system, blocking cell lysis induced by bacterial toxins and Zellweger cerebro‐hepato‐renal syndrome^22,51^. Let alone the characteristic traits of linoleic acid as anti-thrombotic, anti-irritant, anti-inflammatory and anti-tumor activities^7^. Interestingly, the collective presence of all such FAs in the profile of *Paradendryphiella *sp., chiefly under cold and prolonged incubation, directed us to investigate their anticancer activity in a comparative manner, for the first time till our knowledge.

As well known, the fundamental target of conventional anticancer treatments, namely chemotherapy and radiotherapy, is to limit tumor growth and block metastasis. Despite their effectiveness, several toxic side effects are reported, which ranged in their severity from simple to chronic and lethal such as tiredness, nausea, hair loss, blood clotting, sterility and congestive heart failure, etc.^52^. However, the development of intrinsic or acquired drug resistance of the tumor cells is the main obstacle of anticancer agents. Therefore, the emergence of adjuvant treatments, that augment anticancer efficacy and attenuate drug resistance simultaneously with low adverse effects, is an urgent solution in cancer therapy^53^. Numerous studies evaluated the antitumor activity of PUSFAs and their conjugation with anticancer drugs and reported promising results via such synergistic interaction^54^.

Herein, the SCOs extracted from *Paradendryphiella *sp. that was produced under psychrophilic/ prolonged incubation displayed the most potentiality against examined cancer cell lines. That could be attributed to the preponderance of PUFAs by 24.20%, relative to that produced at 28 °C either after 8 or 30 days of incubation by 7.15% and 16.03%, correspondingly. Accumulating evidence confirms the potent apoptosis-mediated anticancer activity of PUFAs on different cancer cells via elevating free radicals with increasing lipid peroxidation and subsequently disintegration of the cell membrane, mitochondria, and nucleus^54–56^. Previous study manifested that the anticancer activity-dependent prooxidant effect of PUFAs was mainly attributed to octadecenoic acid^56^. Obviously, SCOs produced under psychrophilic/ prolonged incubation contained the highest percentage of octadecenoic acid which is one of the most widely distributed PUFAs in natural oils. This fatty acid has highly effective potential for eradicating several human cancer cells (lung, breast, pancreases, liver, and leukemia)^56–58^. Moreover, in comparison with the others, SCOs produced under psychrophilic/ prolonged incubation included the highest content of ω 6-PUFA (23.53%) which is 35.4-fold higher than ω 3-PUFA. ω6-PUFA inhibited tumor cell proliferation more effectively than ω3-PUFAs, making it a promising agent for cancer therapy^54^. Aside from increasing the cellular content of reactive oxygen species and lipid peroxidation^59^, ω6-PUFAs suppressed oncogenes and enhanced proapoptotic gene expression with halting cell cycle progression^54^. Previous studies reported that ω6-PUFAs (linoleic acid) upregulated p21 gene expression and downregulated cyclins D and A expression^60,61^. Other previous studies illustrated the suppressive effect of ω6-PUFAs (linoleic acid) on BCl2 and NF-κB gene expressions with enhancing BAX expression in the treated cancer cell lines^62,63^. In the line with the above-mentioned previous studies, SCOs produced under psychrophilic/ prolonged incubation, which had the highest content of PUFAs (particularly, ω6) and exhibited the maximum potential for increasing cellular lipid peroxidation, mediated the highest potential for increasing the expression of key apoptotic genes and decreasing oncogene expression.

In a word, the present study succeeded in producing SCOs with eminent FAs profile that displaying distinguished anticancer traits through recruiting low cost agro-industrial residue and seawater as nutritive and aqueous media, respectively. Remarkably, the capability of oleaginous *Paradendryphiella sp*., by the dint of its marine origin, to grow and accumulate lipids, under acidic on a par with alkaline conditions, encourage harnessing other Egyptian marine sources, with different acidity/alkalinity nature such as Siwa lakes (pH ≤ 4) and Wadi El-Natrun Salt Lakes (pH ≥ 9), as aqueous medium for propagation of oleaginous *Paradendryphiella *sp. In addition, the significant growth and remarkable lipogenesis process under different temperatures highlight the compatibility and adaptability of *Paradendryphiella *sp. to cope with seasonal variation (i.e., winter and summer). Interestingly, the cultivation of fungal biomass in high inoculum size under cold-acidic conditions would exclude the step of media sterilization; denoting save in time and energy especially during fermentation process. As stated by^64,65^, low pH level of 4.0 could handicap the growth of undesired contaminations in non-sterile medium, in particular during lipid production by cold-adapted fungi.

From this, it can be inferred that the physiological features of marine *Paradendryphiella *sp. of the current study shed the light to the feasibility to invest its growth and synthesis of lipids/SCOs using varied marine sources in cheap nutritive media under static conditions and diverse incubation temperature; avoiding by such way the electricity requirement for sterilization, agitation or temperature modulation. Subsequently, for future large-scale reactor operations, the whole process in our study would eliminate the requirement for large amount of fresh water, costly stirring, aeration, and temperature control systems; representing a promising economic strategy convenient for industrial and commercial production. Ultimately, this study fulfilled all aspects of blue economy beginning from proper selection of isolation location; passing through adequate employing of cost-effective nutritive media and economic incubation conditions ending with efficient, biosafe and biocompatible product with a prominent anticancer potentiality.

## Conclusions

To sum up, the present study provided a novel approach for the production of biosafe and proficient anticancer agent, against A549, MDA-MB 231 and HepG-2 cell lines, in an economic process. A successful isolation of *Paradendryphiella *sp. from Egyptian marine resource boosted all the aspects of blue economy. Virtually, 5.5 g/L with 40.7% lipid content was produced under statistically optimized conditions using agro-industrial waste in sea water as an aqueous media. The fungus possess the ability to accumulate SCOs under alkaline, acidic, mesophilic and psychrophilic conditions. The variation in FAs profiles under different conditions encourages their applications as anticancer agent and food supplement in in Vivo ongoing studies. Additionally, their combination with other medications or nanomaterials considers being a promising key for future perspectives.

## Methods

### Samples collection, screening and isolation of marine fungi

Initially, about 6 marine samples were collected, during the period of March 2021 to February 2022, from different locations, along Mediterranean Sea shore, Alexandria, Egypt, at 50–150 cm depth below the sea surface. The water and sediment samples were collected from the east of Alexandria (i.e., Abu Qir, 20 kms east of Alexandria), passing through the middle location (i.e., El-Shatby) ending with the west part of north cost (i.e., El Alamein, 106 kms west of Alexandria). All samples were transported in sterile polyethylene screw-cap bottles in ice box and immediately processed for the isolation of fungi. About 1 ml of each collected sample was plated on Potato Dextrose Agar medium (PDA) (potato extract 4 g/L, dextrose 20 g/L, 10% tartaric acid 14mL, Agar 15 g/L, pH 5.4 ± 0.4) and then incubated at 28 ºC for 3–6 days^66^. After incubation, the plates were observed for the development of distinct fungal colonies, which were individually picked off, purified and maintained on PDA slants at 4^∘^C.

### Screening for oleaginous fungi, dry weight determination and quantification of total lipids

Primarily, the procured fungal isolates were checked for intracellular lipid accumulation using Nile- red staining assay as described previously in details by^66^. Accordingly, the selected oleaginous fungal isolates with the highest fluorescence signals were cultivated on lipid producing medium containing the following ingredients (g/L), molasses 30.0, and yeast extract 2.0, PH 6. About 50 ml of medium dissolved in seawater was dispended in 250 ml flasks in triplicate and incubated at 28 °C with a shaking speed of 120 rpm for 7 days. After incubation, dry biomass, lipid yield and content were determined as described briefly in^67^. The fungal isolates that exhibited the highest lipid content was selected for further steps.

### Morphological and molecular identification of the selected fungal isolate

The cultural, microscopic and 18S rRNA gene sequencing was utilized to characterize and identify the selected isolate, which actively grew for up to 5 days on the PDA plate at 28 °C. The cultural properties, which include colony shape, size, color and texture, were investigated; the microscopic properties were depicted by light and scanning electron microscope (SEM). However, molecular identification was carried out by using 18S universal primer pairs of ITS1 (5′-TCCGTAGGTGAACCTGCGG-3′) and ITS4 (5′-TCCTCCGCTTATTGATATGC-3′)^68^. The amplified PCR product was sequenced by an ABI 3730 automated sequencer (PerkinElmer/Applied Biosystems (Foster City, CA, USA). To assess similarity, the BLASTn analysis was used; thereafter, the corresponding accession number was obtained; the evolutionary analysis was conducted by MEGA- 6 software package using the neighbor-joining (NJ) approach with bootstrap analyses for 1000 replicates.

### Optimization of nutritional and incubation parameters

#### Central composite design

In order to maximize SCOs productivity, five levels (− 2, − 1, 0, + 1, + 2) of four different media components (i.e., molasses concentration, yeast extract concentration, incubation time and initial pH) were studied as listed in Table (1). In 250 mL Erlenmeyer flasks containing 50 mL of medium, each trial in 31 test matrix was prepared, inoculated and incubated statically at 28 °C. Each trial was carried out in triplicates and lipid yield as a response was calculated. The lipid yield was obtained following lipid extraction and esterification by protocol described in details by^69^. For statistical calculation, the relationship between the coded and actual values is described by Eq. (2):

2 $$ {\text{Xi}} = {\text{Ui}} - {\text{Ui}}0/\Delta {\text{Ui}} $$

where *Xi* is the coded value of the *i*th variable, *Ui* is the actual value of the *i*th variable, Ui_0_ is the actual value of the *i*th variable at the center point and ΔUi is the step change of variable. The response variable (lipid content) suitable to a quadratic equation for the variables was as Eq. (3):

3 $$ \begin{aligned} & {\text{Y}} = \beta 0 \, + \, \beta {\text{1X1 }} + \, \beta {\text{2X2 }} + \, \beta {\text{3X3 }} + \, \beta {\text{11X12}} + \, \beta {\text{22X22 }} \\ & \quad + {\kern 1pt} \, \beta {\text{33X32}} + \, \beta {\text{12X1X2 }} + \, \beta {\text{13X1X3 }} + \, \beta {\text{23X2X3}} \\ \end{aligned} $$

where *Y* is the predicted response; X_1_, X_2_, X_3_ are input variables which influence the response variable Y*; β*_0_, intercept; *β*_1_, β_2_ and *β*_3_ linear coefficients; *β*_11_, *β*_22_ and *β*_33_, squared or quadratic coefficients *β*_12_, β_13_, and *β*_23_ interaction coefficients.

### Statistical analysis and verification

The statistical software Minitab 15.0 (Minitab Inc., Pennsylvania, USA software) was used to enquiry statistical analysis of data of CCD (matrix design, regression analysis and ANOVA). Further, the relationship between the response and factors was also illustrated by three-dimensional surface plots and two-dimensional contour plots. In addition, the optimizer tool was used to predict the optimum level of experimental factors. Under predicted optimized conditions, the model was validated through comparing lipid yield with that obtained from basal conditions^69^.

### The effect of abiotic factors on fatty acid profile

The effect of prolonged incubation time, temperature and also pH on lipid composition were investigated. In sterile optimized media, the flasks were incubated at 10 °C and 28 °C for 8 days and 30 days to study the effect of temperature and time, respectively. However, other flasks contained optimized medium were adjusted to pH 3 and 9 and incubated at 28 °C, to examine the influence of pH. In all these trials, the biomass was harvested to determine fatty acid composition as would be described posteriorly in the next section.

### Lipid extraction, transestrification and determination of SCOs profile using Gas chromatography

Extraction and transestrification of lipids were performed as briefly described in^67^. The transesterified lipid, produced by the selected fungal strain under optimized conditions and also under different abiotic factors, were analyzed by Agilent 6890 gas chromatograph equipped with a straight deactivated 2 mm direct injector liner and a 15 m Alltech EC-5 column (250 μ I.D., 0.25 μ film thickness), to detect and quantify saturated and unsaturated contents of fatty acid. The operating conditions were held at 250 °C for inlet temperature, 280 °C for detector temperature and 35 °C initial oven temperature, which was held for 2 min then elevated to 300 °C for 23 min. The injection volume was 2 mL, with a split ratio of 10:1. Helium served as a carrier gas at a constant flow rate of 1 ml/min. The FA profile was identified via comparison of its chromatographic peaks and retention times with those of WILEY 09 and NIST 11 mass spectral database. Notably, each individual peak was quantified by means of standards and their corresponding calibration curves^68^.

### Determination of cytotoxicity of extracted SCOs on normal human cell line

Normal human lung fibroblast Wi-38 cell line (CCL-85) was used to detect cytotoxicity of the studied lipids (obtained from optimized media and after incubations for 30 days at 10 °C and 28 °C). DMEM medium (Lonza, USA) containing 10% fetal bovine serum (GIBCO, USA) was used to culture the Wi-38 cell line. A 96-well cell culture plate was seeded with 5 × 10^3^ cells per well, and the cells were then incubated at 37 °C in a 5% CO_2_ incubator. Serial lipid concentrations samples were incubated with Wi-38 cells for 72 h after 24 h had passed for cell attachment. The MTT assay was used to assess normal cell viability^70^. The wells were loaded with 20 µl of 5 mg/ml MTT (Sigma, USA), and the plate was then incubated at 37 °C for 3 h. Following the removal of the MTT solution, 100 µl of DMSO was added, and the absorbances of wells were assessed at 570 nm using a microplate reader (BMG LabTech, Germany). The studied lipids' effective safe concentration (EC_100_) value (at 100% cell viability) was calculated.

### Investigating the cytotoxicity of the studied SCOs against cancerous human cell lines

The above-mentioned lipid samples' anticancer activity was evaluated utilizing three cancer cell lines. Lung cancer cell line (A549, CCL-185^TN^), triple negative breast cancer cell line (MDA-MB 231, HTB-26), and liver cancer cell line (HepG-2, HB-8065) were supplied from the American Type Culture Collection (ATCC, USA). These tested cell lines were cultured in DMEM with 10% fetal bovine serum. In sterile 96-well cell culture plates, all cancer cells (4 × 10^3^ cells/well) were seeded. After 24 h, serial amounts of the evaluated lipid samples were added to cells and incubated for 72 h at 37 °C in a 5% CO2 incubator. The MTT technique was used as previously mentioned. The half maximum inhibitory concentrations (IC_50_) were estimated using GraphPad Prism. Furthermore, the morphological changes in cells before and after treatment with the extracted lipids were observed using the phase contrast inverted microscope with a digital camera (Olympus, Japan).

### Determination of cellular lipid peroxidation level in the treated cancer cells

In order to deduce the mechanism of anticancer performance triggered by the examined lipids, the over-generation of lipid peroxidation products were measured. The lipid peroxidation assay was performed using thiobarbituric acid reactive substance assay as described by^71^. Briefly, after 72 h of incubating the extracted lipids with cancer cell lines, supernatants of both treated and untreated cells were collected. About 100 μl of supernatant was incubated eventually with thiobarbituric acid (0.67%) and trichloroacetic acid for 30 min in boiling water bath. After that, the mixture was centrifuged and the supernatant was measured at 532 nm. The lipid peroxidation level was estimated by standard curve of malondialdehyde. The analysis was done for three measurements and the data are expressed as mean ± standard error of mean (SEM) and the significant values were considered at *p* < 0.05. One-way analysis of variance (ANOVA) by Tukey’s test used for evaluating the difference between the mean values of the studied treatments.

### Real-time quantitative PCR analysis for oncogenes and proapoptotic genes

Lung cancer cell line (A549) was incubated with the most effective SCOs, at IC_50_, for 72 h in 5% CO_2_ incubator. RNAs of untreated and treated cancer cells were extracted using Gene JET RNA purification kit (Thermo Scientific, USA). Then cDNAs were synthesized using cDNA Synthesis Kit (Thermo Scientific, USA). Real time PCR was performed using SYBR green master mix and specific primers (Forward/Reverse) as shown in Supplementary Table S7. The thermal cycling parameters were pre-denaturation, followed by 40 amplification cycles of 1 min at 95 °C and 30 s at 60 °C and at 72 °C for 30 s. GAPDH mRNA was quantified to adjust the amount of mRNA in each sample. The 2^−ΔΔCT^ equation was used to estimate change in gene expressions before and after treatment of cancer cells.

### Supplementary Information

Supplementary Information.

## Data Availability

The datasets analyzed during the current study are available in https://www.ncbi.nlm.nih.gov/search/all/?term=OQ134928 with accession number (OQ134928). All data generated or analyzed during this study are included in this published article.

## Abbreviations

SCOs: Single cell oils
PUFAs: Poly unsaturated fatty acids
NR: Nile-red staining technique
PDA: Peptone dextrose agar
NJ: Neighbour-joining approach
CCD: Central composite design
R^2^: Coefficient of determination
Adj-R^2^: Adjusted-R^2^
ANOVA: Analysis of variance
USFAs: Unsaturated fatty acids
SFAs: Saturated fatty acids
GC: Gas chromatography
MUSFAs: Monounsaturated fatty acids
FAs: Fatty acids
SEM: Standard error of mean
FBS: Fetal bovine serum
