# Supplementary material for: Unveiling the anticancer potentiality of single cell oils produced by marine oleaginous Paradendryphiella sp. under optimized economic growth conditions
Source: Sci Rep. 2023 Nov 26;13:20773. doi: 10.1038/s41598-023-47656-x (PMC10679151; doi:10.1038/s41598-023-47656-x)
Supplement: Supplementary file 1 — Supplementary Information. [file 41598_2023_47656_MOESM1_ESM.docx]

**Unveiling the anticancer potentiality of single cell oils produced by marine oleaginous *Paradendryphiella sp.* under optimized economic growth conditions**

**Hadeel El-Shall^1^, Marwa Abu‑Serie^2^, Gadallah Abu-Elreesh^1^, Marwa Eltarahony^1*^**

^1^ Environmental Biotechnology Department, Genetic Engineering and Biotechnology Research Institute (GEBRI), City of Scientific Research and Technological Applications (SRTA-City), 21934, New Borg El-Arab City, Alexandria, Egypt

^2^ Medical Biotechnology Department, Genetic Engineering and Biotechnology Research Institute, (GEBRI), City of Scientific Research and Technological Applications (SRTA-City), 21934, New Borg El-Arab City, Alexandria, Egypt

* **Corresponding author.**

**E-mail:**

meltarahony@srtacity.sci.eg **(**Marwa Eltarahony**)**

[m_eltarahony@yahoo.com](mailto:m_eltarahony@yahoo.com)

Supplementary Table S1 Dry weight, lipid yield and lipid content estimations for screened isolates

| **Isolate** | **Dry cell weight (g/L)** | **Lipid yield(g/L)** | **Lipid content (%)** |
| --- | --- | --- | --- |
| **H1** | 4.47 ± 0.12 | 0.85 ± 0.0005 | 19.01 |
| **H2** | 8.5 ± 0.14 | 1.76 ± 0.002 | 20.7 |
| **H3** | 11.82 ± 0.05 | 2.61 ± 0.03 | 22.08 |
| **H4** | 10.39 ± 0.09 | 1.91 ± 0.06 | 18.38 |

Supplementary Table S2 ANOVA for quadratic polynomial model of lipid content extracted from *Paradendryphiella sp.*

| **Soure** | **DF** | **Seq SS** | **Adj SS** | **Adj MS** | **F** | **P** |
| --- | --- | --- | --- | --- | --- | --- |
| **Regression** | 14 | 40.7036 | 40.7036 | 2.9074 | 35.6 | 0 |
| **Linear** | 4 | 14.0285 | 14.0285 | 3.50712 | 42.95 | 0 |
| **Square** | 4 | 22.4731 | 22.4731 | 5.61829 | 68.8 | 0 |
| **Interaction** | 6 | 4.202 | 4.202 | 0.70033 | 8.58 | 0 |
| **Residual error** | 16 | 1.3065 | 1.3065 | 0.08166 |  |  |
| **Lack of fit** | 10 | 0.7759 | 0.7759 | 0.07759 | 0.88 | 0.593 |
| **Pure Error** | 6 | 0.5307 | 0.5307 | 0.08845 |  |  |
| **Total** | 30 | 42.0101 |  |  |  |  |

**Supplementary Fig. S3**: Response optimizer for optimum concentrations of examined independent variables for maximum productivity of SCOs from marine oleaginous *Paradendryphiella sp.*


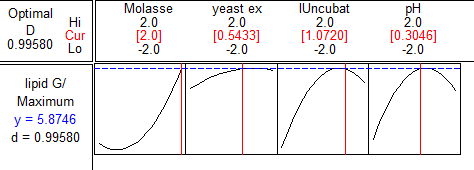


**Supplementary Fig.S4 Dose response curves** of SCOs for inhibiting growth of (A) A549, (B) MDA-MB 231 and (C) HepG2 cells after 72 h incubation. All values are demonstrated as mean ± SEM.


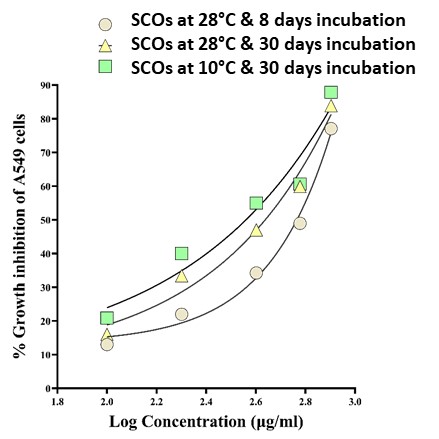


**A**


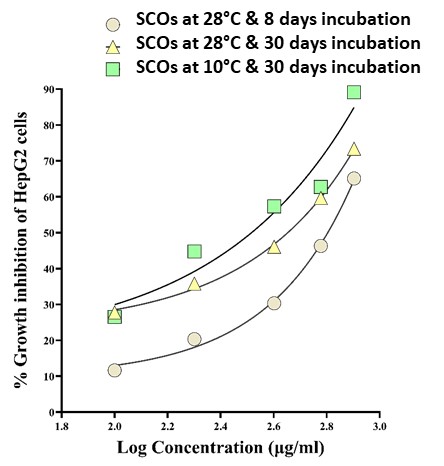


**C**


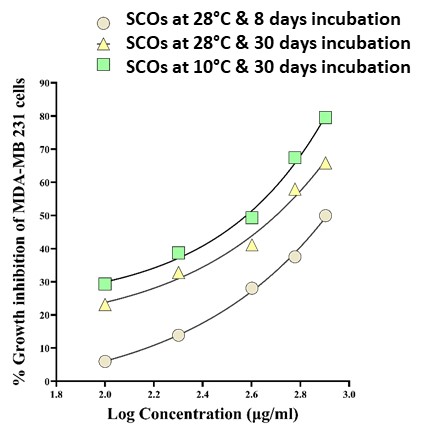


**B**

Supplementary Table S5: Relative fold increment in lipid peroxidation

| **Sample** | **A549** | **MDA-MB 231** | **HepG2** |
| --- | --- | --- | --- |
| **SCOs at 28°C & 8 days incubation** | 1.854±0.200 | 1.23±0.104 | 2.59±0.454 |
| **SCOs at 28°C & 30 days incubation** | 3.249±0.395 | 1.869±0.185 | 4.844±0.283 |
| **SCOs at 10°C & 30 days incubation** | 7.796±0.746 | 3.911±0.651 | 10.66±0.403 |

All values are expressed as mean ± SEM

Supplementary Table S6: The relative fold change in gene expression of A549 cells treated with SCOs produced under psychrophilic/ prolonged incubation

| **Gene** | **Relative fold change** |
| --- | --- |
| NF-κB | 0.094±0.006 |
| BCl2 | 0.204±0.042 |
| Cyclin D | 0.130±0.021 |
| Bax | 5.35±0.755 |
| p21 | 3.72±0.688 |

All values are expressed as mean ± SEM.

Supplementary Table S7: Primer sequences of oncogenes and proapoptotic genes

| **Gene** | **Primers** |
| --- | --- |
| **NF-κB** | Forward: 5′-TCAAGATCTGCCGACTGAAC-3′  Reverse: 5′-CCTCTTTCTGCACCTTGTCA-3′ |
| **BCl2** | Forward: 5′-CTGGTGGACAACATCGCCCT-3′  Reverse: 5′-TCTTCAGAGACAGCCAGGAGAAAT-3′ |
| **Cyclin D** | Forward: 5′-TACTCTGGCGCAGAAATTAGGTC-3′  Reverse: 5′-CTGTCTCGGAGCTCGTCTATTTG-3′ |
| **Bax** | Forward: 5′-CCGCCGTGGACACAGAC-3′  Reverse: 5′-CAGAAAACATGTCAGCTGCCA-3′ |
| **P21** | Forward: 5′-CTGGGGATGTCCGTCAGAAC-3′  Reverse: 5′-5′-GCCATTAGCGCATCACAGT-3′ |
